# Global differential expression of genes located in the Down Syndrome Critical Region in normal human brain

**Published:** 2014-12-30

**Authors:** Julio Cesar Montoya, Dianora Fajardo, Angela Peña, Adalberto Sánchez, Martha C Domínguez, José María Satizábal, Felipe García-Vallejo

**Affiliations:** 1 Department of Physiological Sciences, School of Basic Sciences, Faculty of Health, Universidad del Valle. Cali, Colombia; 2 Laboratory of Molecular Biology and Pathogenesis LABIOMOL. Universidad del Valle, Cali, Colombia; 3 Faculty of Basic Sciences, Universidad Autónoma de Occidente, Cali, Colombia

**Keywords:** Brain, cerebral cortex, homeostasis, Down Syndrome critical region, oligonucleotide array sequence analysis, transcriptome, gene expression profiling

## Abstract

**Background:**

The information of gene expression obtained from databases, have made possible the extraction and analysis of data related with several molecular processes involving not only in brain homeostasis but its disruption in some neuropathologies; principally in Down syndrome and the Alzheimer disease.

**Objective:**

To correlate the levels of transcription of 19 genes located in the Down Syndrome Critical Region (DSCR) with their expression in several substructures of normal human brain.

**Methods:**

There were obtained expression profiles of 19 DSCR genes in 42 brain substructures, from gene expression values available at the database of the human brain of the Brain Atlas of the Allen Institute for Brain Sciences", (http://human.brain-map.org/). The co-expression patterns of DSCR genes in brain were calculated by using multivariate statistical methods.

**Results:**

Highest levels of gene expression were registered at caudate nucleus, nucleus accumbens and putamen among central areas of cerebral cortex. Increased expression levels of RCAN1 that encode by a protein involved in signal transduction process of the CNS were recorded for PCP4 that participates in the binding to calmodulin and TTC3; a protein that is associated with differentiation of neurons. That previously identified brain structures play a crucial role in the learning process, in different class of memory and in motor skills.

**Conclusion:**

The precise regulation of DSCR gene expression is crucial to maintain the brain homeostasis, especially in those areas with high levels of gene expression associated with a remarkable process of learning and cognition.

## Abstract (es)

**Introducción:**

La información de la expresión de genes consignada en bases de datos, ha permitido extraer y analizar información acerca procesos moleculares implicados tanto en la homeostasis cerebral y su alteración en algunas neuropatologías.

**Objetivos:**

Correlacionar los niveles de transcripción de 19 genes localizados en la región crítica del cromosoma 21, asociada a Síndrome de Down (DSCR), con la localización cerebral y su coexpresión en diferentes subestructuras del cerebro humano.

**Métodos:**

A partir de valores de expresión génica disponibles en la base de datos del proyecto cerebro humano del Atlas del Cerebro del "Allen Institute for Brain Sciences" (http://human.brain-map.org/), se construyeron perfiles de expresión de 19 genes DSCR en 42 subestructuras cerebrales. Además, utilizando métodos estadísticos multivariados se analizaron los patrones de coexpresión de genes DSCR en el cerebro normal.

**Resultados:**

En el núcleo caudado, el núcleo accumbens y el putamen además de las Áreas centrales 2, 3 y 4, se determinaron los valores de expresión más elevados para los genes incluidos RCAN1, que codifica para una proteína involucrada en el proceso de transducción de señales de SNC; PCP4 cuya proteína interviene en la unión a la calmodulina y TTC3 una proteína que interviene en la diferenciación de neuronas. Las subestructuras identificadas con una elevada expresión de estos genes, están asociadas con procesos de aprendizaje, en diferentes tipos de memoria y habilidades motoras.

**Conclusiones:**

La regulación de la expresión de los genes DSCR es clave para mantener la homeostasis cerebral, especialmente en aquellas áreas de mayor expresión, las cuales están asociadas con procesos sumamente importantes.

## Introduction

The brain is the system responsible for a wide variety of biological processes ranging from simple motor functions to complex cognitive processes. Among all anatomical areas that comprise the brain, it is possible to highlight the basal nuclei and the structures that are part of the limbic system, highly related to motor functions as well as in learning and memory processes 1.

The cerebral homeostasis is measured by complex interactions that must be carried out between the great abundance and diversity of neurons, glial cells and non-neuronal cells that comprise it. This derives from an accurate gene regulation in all structures 2. It has been observed that approximately 30% of all human genes express mainly in this organ. Besides, that cerebral level of transcription have a marked variability that depends on the analyzed area, associated to groups of genes co-expressing mainly in certain brain areas 3. However, most studies have been conducted on homogenate tissues, having to discern about the cellular heterogeneity and the functionality of such genes in those regions.

The abnormal variation in gene dosage can lead to a number of disorders that produce, among other things, neurological class dysfunctions. Thus, Down syndrome (DS) is caused by partial or total triplication of the genes found on chromosome 21, being the most frequent aneuploid which produces varying degrees of cognitive disability 4 ^-^ 5. Studies on partial trisomies have allowed the characterization of a region of the chromosome 21 known as Down Syndrome Critical Region (DSCR). It is located at the distal end of the long arm of chromosome 21 (21q22.1-22.3), and has candidate genes whose imbalance may induce a marked cognitive deficit, as well as other pathologies and associated conditions 6.

Although the involvement of DSCR as the sole cause of DS symptomatology is still a topic of debate, different studies have suggested that this region plays a primary role in the genetic interactions that could be related to the pathogenesis of DS 7. However, the subset of genes that are over expressed on chromosome 21 and generate these deficiencies, are not completely known 8; moreover, some of them do not over express in the trisomic fetal brain 9. For this reason, a detailed knowledge of the expression DSCR genes in normal human brains, could provide a greater understanding of their participation on the cognitive processes while, at the same time, more will be known about their implications in both, neurological dysfunctions associated with DS as in other neuropathologies such as Alzheimer disease.

Some methodologies have been implemented to study the cerebral transcriptome. One of them is the DNA microarrays that allow the analysis of many tissues by measuring the gene expression. In this way, some comparative studies among expression profiles of different brain zones have been accomplished in order to better understand, the physiopathology of many diseases 10 ^-^ 11. However, in this sense, there are several constraints, ranging from the collection of a significant number of brains from donors with no neuropsychiatric and/or neuropathology history, to the correct extraction of brain tissue and optimum quality of RNA in all structures.

In this scenario, bioinformatics databases allowed to extract complete and detailed information of the levels of gene transcription from several brain structures obtained from rigorous computational tools as well as from experiments using microarrays of DNA of postmortem human brains. 

The Allen Institute of Brain Science has compiled a public database called Allen Human Brain Atlas (http://www.alleninstitute.org) that includes anatomic measurements of gene expression and genomic information of human brains. It also has a set of visualization tools and data mining including a complete coverage from histological preparations and microarray data with more than 62,000 probes being possible to obtain transcriptional data of thousands of genes referentiated with Entrez Gene Encode. At least two different probes are available for 93% of 21,245 from the genes consigned.

We designed a bioinformatics model of the expression profiles of 19 genes located at DSCR in 24 specific substructures of cerebral nuclei and 18 from the limbic lobe of normal human brain. Data of brain gene expression coming from DNA microarray experiments are available at the free access database of the human brain project of the Brain Atlas of "Allen Institute for Brain Sciences" in Seattle, Washington (http://www.brain-map.org). The results conducted us to approach to a systemic model of expression that can be bioinformatically modified to extrapolate it with other brain pathologies, thus providing a powerful tool for its understanding.

## Materials and Methods

###  Type of study

This descriptive bioinformatics study (*in silico*), used the Allen Human Brain Atlas free online database (http://human.brain-map.org), which is supported by the Allen Institute for Brain Sciences. The expression level of 19 DSCR genes (21q22.12-22.2) was studied in the cerebral nuclei and the limbic lobe.

### Data mining

With the help of the graphic cursor from the electronic page of the human brain project from the Allen Institute for Brain Sciences, the gene expression level values, along the different substructures of the limbic lobe and the cerebral nuclei, were identified and registered as well as the standard data (z-score). These values were entered into electronic Excel sheets for further analysis. For each one of the substructures included in the study, three values were taken at three different points of each brain substructures considering as data the correspondent z-score mean. The z-score had normalized value in ranges from -3 to +3. Additionally, genomic information was extracted from Gene Ontology application (GO) (http://www.geneontology.org/index.shtml *). *The cellular location, the molecular function and the biologic process of the selected genes were itemized.

### Calculating the Z-score

We consider appropriate to describe in a general way the experimental procedures that led us to obtain the registered z-score data. The procedures are reported extensively on the technical report of The Allen Institute of Brain Science.

The z-score values were calculated by subtracting the total average intensity of the genes in the microarray from the intensity of each individual gene within a single experiment and dividing the result by the standard deviation (SD) of the measure of all the intensities in accordance with the following formula: Z score= (G intensity - average intensity of G1. . .Gn)/DSG1. . .Gn. Where G is a gene within the microarray and G1. . . Gn represents the aggregate measure of all genes in the microarray 12.

### Selection of genes and brain structures

The main inclusion criteria that was followed to select the nineteen genes included in this study was the linkage of the gene product with clinical manifestations of Down syndrome (Table 1). Structures of the cerebral nuclei and the limbic lobe were chosen because they showed higher expression in a preliminary study performed with eight genes of DSCR 13. The main information of each one of the nineteen genes is described in Table 2.

**Table 1.**
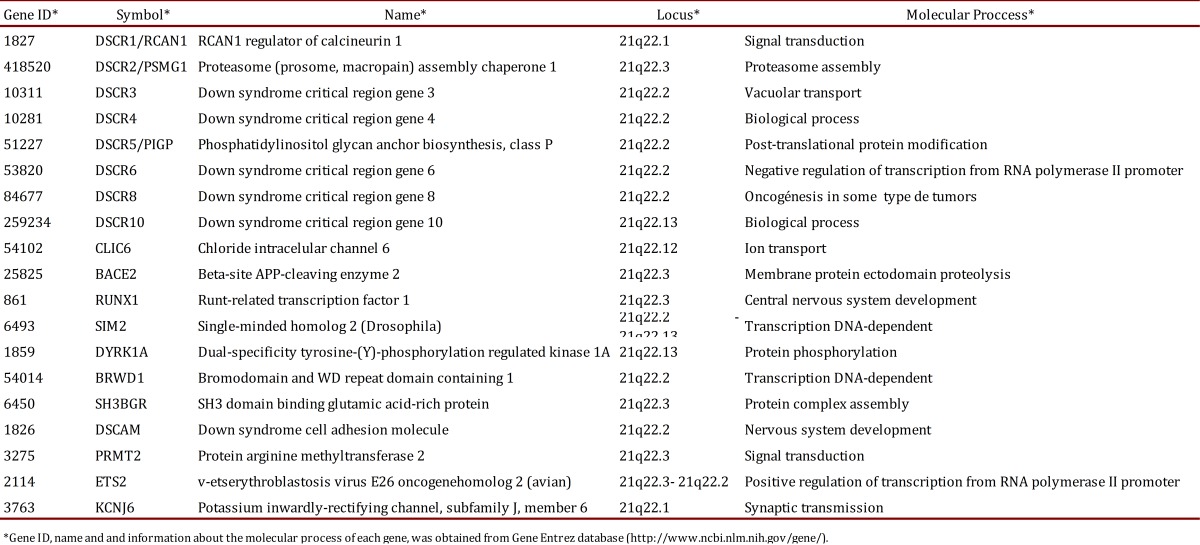
Summary of the protein-coding genes located at HSA21/DSCR including in the present work. Gene ID, name and and information about the molecular process of each gene, was obtained from Gene Entrez database (http://www.ncbi.nlm.nih.gov/gene/).

**Table 2:**
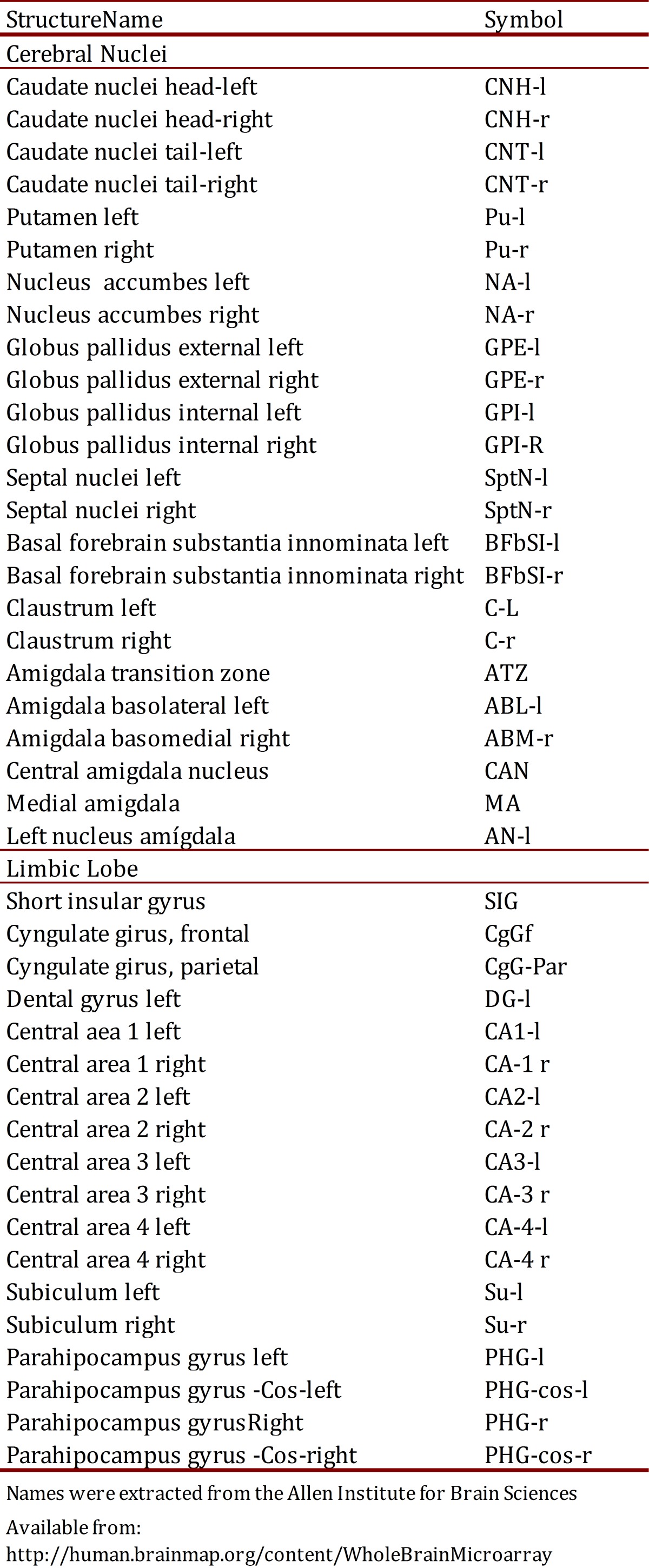
Names and symbols of different structures of Limbic Lobe and Cerebral Nuclei. The names were extracted from the Allen Institute for Brain Sciences.

### Statistical analysis

Statistical analyses were performed using STATGRAPHICS Centurion XVI (http://www.statgraphics.com) and Systat 13 programs (http://www.systat.com). The differences between the data were calculated using a multiple comparison analysis estimating its significance with the Mann and Whitney test for multiple comparisons. Differences in expression levels per gene and anatomical area were modeled through the scatter plot using as comparison measure the Pearson partial correlation values with a significance of 95% (*p* <0.05). The multiple regression analysis of paired comparisons was carried out using the Bayesian regression calculation. To rank the expression in several brain substructures of cerebral nuclei and limbic lobe for each one of genes a Principal Components Analysis (PCA) was performed reducing the R space from 19 coliner variables to 6 principal components (R^19^>R^6)^; the PCA analysis was complemented with a Cluster Analysis (CA).

## Results

### Differential expression of DSCR in human brain

**Figure 1.**
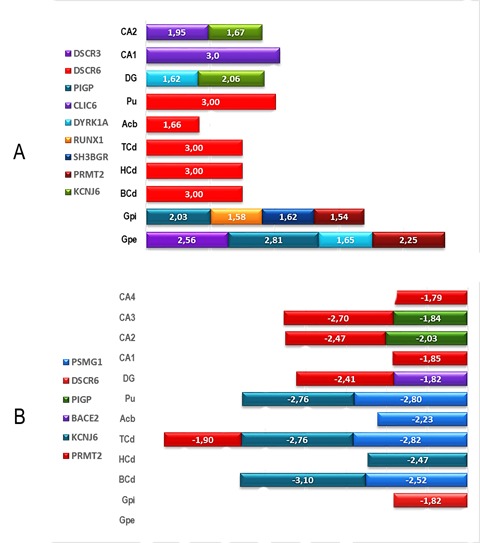
Global expression profiles of nineteen genes located at the HAS21/DSCR along different sustructures of cerebral nuclei and limbic lobe. (A) Genes overexpressed per brain substructure. (B). Genes underexpressed per brain substructure. The mean z-score of DSCR genes was obtained from transcription data of several DNA microarrays consigned in the public database of the Allen Human Brain Atlas (http://www.alleninstitute.org). Caudate nuclei head-left (CNH-l), Caudate nuclei head-right (CNH-r), Caudate nuclei tail-left (CNT-l), Caudate nuclei tail-right (CNT-r), Putamen left (Pu-l), Putamen right (Pu-r), Globus pallidus external left (GPE-l), Globus pallidus internal (GPI), Dental gyrus (DG), Central area 1 (CA1), Central area 2 (CA2), Central area 3 (CA3) and Central area 4 (CA-4).

A quantitative analysis of global transcription of DSCR genes along the structures of the brain nuclei and the limbic lobe revealed that the highest values of z-score were registered in the area that includes the putamen for DSCR3, RCAN1 as well as in left and right caudate nuclei for DSCR6, and the left and right globus pallidus for DSCR3, SH3GBR, DYRK1A, CLIC6 and PRMT2. Moreover, in the limbic lobe, the highest expression values were registered at DG for DYRK1A and KCNJ6 and in the CA1 and CA2 for DSCR3 and KCNJ6 (Fig. 1A). In contrast, the lowest values of expression were registered in basal caudate nuclei for KCNJ6, PSMG1; in CA3, and CA4 for PIGP, DSCR6 and PRMT2 (Fig. 1B).

### Expression in cerebral nuclei

The RCAN1 gene showed high levels of expression in the left putamen (z-score 2.87), the PUr (2.66) and in the left external globus pallidus (2.67); whereas in the C-l, it was registered a lowest level of expression (-1.268). The DSCR2 was not over-expressed in any of the structures of this brain area; however, it is important to highlight that from the all structures of cerebral nuclei, the Pu-l, the Pu-r and the GPE-l, were the areas with lower transcription levels. The DSCR3 showed low expression overall the structures, however, in the GPE-l the highest value of z-score was registered (1.089) in contrast with the ABL-l where a low level of expression was recorded (-1.28).

DSCR4 registered a high z-score in nucleus accumbens -NA- (1.84) and in Pu-r (1.40). In the nucleus of the left amygdala, the z-score was -1,022. PIGP had the highest expression in the Pu-r (1.97). DSCR6 was variable levels of expression in the different structures of cerebral nuclei. However, the highest z-score values were for the Pu-l (2.95); in CNH-l (2.04); CNH-r (2.31) and CNT-r (2.62). The highest-score of DSCR8 (1.46) was observed in the Pu-l. DSCR10 had high levels of expression in CN-r (2.58) and in GPE-r (2.16), whereas ATZ had the lowest z-score (-1.72).

BACE2 and ERG showed a maximum level of expression in the external and internal left globus pallidus (2.44/2.09 and 2.37/2.22) respectively. BACE2 also had over-expression in the GPI-r (2.176). DSCAM, DYRK1A, KCNJ6 had low expression levels in the caudate nucleus (-1.94/-1.86/-2.28). DYRK1A and KCNJ6 also presented under expression in GPE-r (-1.69/-1.57). Additionally, KCNJ6 registered low expression in the left (-2.40) and right (-2.16) putamen and left internal and external globus pallidus (-1.78/-1.53). RUNX1 and SIM2 presented maximum under expression in the nucleus of the amygdale (-1.93/-1.62). Moreover, RUNX1 registered mid values of over expression in CN, Pu and GP; BRWD1, CLIC6, ETS2, and SH3BGR had low levels of expression.

### Expression in the limbic lobe

In CA4-l the highest values of z-score for DSCR1, 2, 3, 4, 5, 6 y 8 were recorded (2.04 ± 0.56). In other structures, variable levels of expression were observed being DSCR1 the one with the lowest level of expression. PIGP showed its highest level of expression in CA4-r (2.09). In the subiculum, DSCR6 recorded a low level of expression (-1.39). CLIC6 had high values of expression in CA1-l (2.807) and in PHG-r (2.147). BRWD1 and DYRK1A registered low levels of expression in the CA 4 (-1.598/-1.506 respectively). BACE2 and RUNX1 showed variable low levels of expression in left dental gyrus (-2.431/-1.355) respectively. On the other hand, ERG, ETS2, SH3BGR, SIM2 had in general low, levels of expression.

### Co-expression of genes located in DSCR along the different regions of the normal human brain

**Figure 2.**
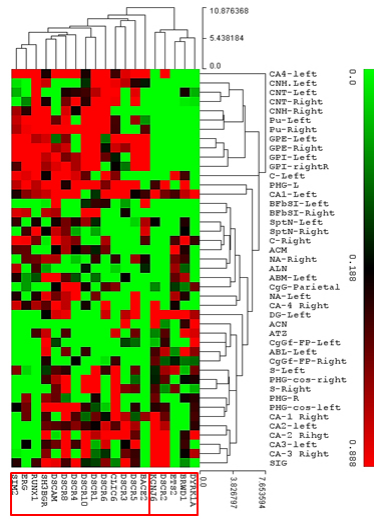
Heath map of the cluster analysis using the medians distance values of nineteen genes of DSCR along several substructures of the Cerebral Nuclei and Limbic Lobe. Color scale indicates that red zones are more related meanwhile the greens are less associated.

A Cluster Analysis (CA), using the median distance values, revealed that the expression of some genes was produced in association with a series of sub structures both from cerebral nuclei and limbic lobe (Fig. 2). For CA3 and DG, a high association in the co-expression of the genes DSCR2, KCNJ6 was determined. In contrast, in the CN and the GP, a high association in the co-expression of RUNX-1, ERG y SIM2 was recorded. In CN, it was possible to define a close association in the co-expression of DSCR1, DSCR6, DCR10 and CLIC-6.

The PCA for the cerebral nuclei showed six components that included 85.6% of the total variance. In the first component, DSCR1, 8, 10, BACE2, ERG and RUNK1 genes were grouped, while in the second component DSCR4 and ETS2 genes were included. For the Limbic Lobe, six components were discriminated that explained 76.1% of the total variance. The first component DSCR1, ERG y RUNK1 genes were grouped; in contrast with the second component in which DSCR4, DSCR 8 and ETS2 were grouped.

**Figure 3.**
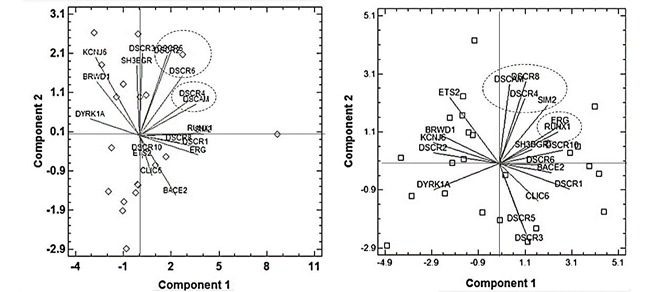
Distribution of nineteen genes located at de DSCR in the two Cartesian planes represented by the biplot obtained using the principal component analysis of the expression values at limbic lobe (left) and cerebral nuclei (right).

The biplots showed two associations for the cerebral nuclei: The first one included the genes DSCAM, DSCR4, DSCR8 and SIM2; while the second included ERG and RUNX (Fig. 3 right). On the other hand, for the limbic lobe the analysis also showed two associations in which the first one included DSCR2, 5 and 6 whereas the second one grouped DSCAM and DSCR4 (Fig. 3 left).

## Discussion

One of the mid-term effects of the Human Genome Project is the large volume of data recorded in different databases making possible its bioinformatics use. The results of these analyses have prompted a new way to understand biological phenomena *in silico* by using different genomic variables recorded in on line databases. In this sense, this study allowed to develop a topographic map of the expression of 19 DSCR genes along different structures of normal human brain. The results obtained evidenced that the transcription of DSCR genes was differential, besides being dependent of the brain-associated structure. Thus, it can be concluded that in a normal brain, the nineteen DSCR genes analyzed, are expressing themselves differentially under a highly precise regulation of its transcription, being this dependent on the functional process of every structural components of human brain.

Previous reports have shown high levels of expression of RCAN1 in cortex, midbrain and cerebellum of mouse DS model 14, and of PIGP gene in the developing brain 15. Moreover, the PIG-P, a gene encoding a component of glicosil fosfatidilinositol-N-acetilglucosaminil transferase (GPI-GnT), was twice over expressed in cerebral cortex, in contrast with DSCR6 whose expression was normal. DSCR4 was not detected in fetal brain 16. Although those previous results evidenced a structure specific differential trancription, our results showed in a more detailed way how the transcription of DSCR genes is particular to each of them and has transcriptional profiles that are dependent upon the brain substructure involved.

Our results showed that RCAN1 and RUNX1 were over expressed in important brain structures associated with several function of learning and cognition, in contrast with DYRK1A that was under expressed in the same brain substructures. Previous results have shown that RUNX1 protein is a heterodimeric transcription factor that binds to the core element of many enhancers and promoters. Its transcription unit located in chr21: 35, 086, 302-35, 343, 066, has a size of 261,498 bp and contains 9 exons; possibly to be involved in the development of normal hematopoiesis 17. Moreover chromosomal translocations involving this gene are well documented and have been associated with several types of leukemia 18. 

The protein encoded by RCAN1, which its transcription unit is located at chr21: 34, 812, 252-34, 909, 180 having a size of 96,929 bp and containing 4 exons, interacts with calcineurin A and inhibits calcineurin-dependent signaling pathways, possibly affecting central nervous system development 19. RCAN1 gene is located in the minimal candidate region for the Down syndrome phenotype, and is overexpressed in the brain of Down syndrome fetuses. Chronic overexpression of RCAN1 may lead to neurofibrillary tangles such as those associated with Alzheimer disease 20.

On the other hand, DYRKA1 which transcription unit is located at chr21: 37, 714, 547-37, 806, 704 has a size of 92,158 bp and contains 11 exons, encoding a member of the dual-specificity tyrosine phosphorylation-regulated kinase (DYRK) family. DYRKA1 contains a nuclear targeting signal sequence, a protein kinase domain, a leucine zipper motif, and a highly conservative 13-consecutive-histidine repeats that catalyzes its autophosphorylation on serine/threonine and tyrosine residues. It may play a significant role in a signaling pathway regulating cell proliferation and may be involved in brain development. It is considered to be a strong candidate gene for learning defects associated with Down syndrome 21.

Moreover, DYRK1A phosphorylates several transcriptional factors, such as CREB and NFAT, endocytic complex proteins, and AD-linked gene products, RCAN1 is an endogenous inhibitor of calcineurin A, and its unbalanced activity is thought to cause major neuronal and/or non-neuronal malfunction in Down syndrome and Alzheimer disease. Interestingly, DYRK1A and RCAN1 contribute to the learning and memory deficit, altered synaptic plasticity, impaired cell cycle regulation, and Alzheimer Disease-like neuropathology in DS. Such results could suggest a potential feedback as an important mechanism of transcription control in these brain areas 22.

A remarkable feature of the present study was the identification of a differential transcriptional control via negative feedback of several genes that depend upon specific brain substructures. One example of it was the overexpression of RCAN1, RUNX1 in contrast with the underexpression of DYRK1A that occur in caudate nuclei head left and right, putamen, internal and external globus pallidus of normal brain Such result, lead us to propose that in normal brain, expression of these genes in specific structures of cerebral nuclei, and limbic lobe regulate not only cognitive function but emotional ones via a complex network of gene expression responsible by the learning and memory functions among others associated process 23 ^-^ 24. Previously it was reported that DYRK1A overexpression and numerous cytosolic, cytoskeletal and nuclear proteins, including transcription factors, are phosphorylated by DYRK1A. Contrasting with underexpression in normal brain described in the present study, DYRK1A overexpression is a central point for the deregulation of multiple pathways in the developing and aging DS brain, with structural and functional alterations including mental retardation and dementia. Moreover, the enhanced phosphorylation of amyloid precursor protein by overexpressed DYRK1A facilitates amyloidogenic amyloid precursor protein cleavage elevating Aβ40 leading to brain β-amyloidosis observed in Alzheimer disease 25.

The putamen is the portion of the basal nuclei that form the most external part of the lenticular nucleus and seems to play an important role in the operative conditioning (learning through reinforcement) 26. The somatosensory and motor cortices, the intralaminar nucleus of the thalamus and the substantianigra, project to the putamen and this, in turn, projects in motor and promotor areas of the cortex through the globus pallidus and the thalamus 27. In addition, the caudate nucleus is one of the components of the basal ganglia located next to the cerebellum, participating indirectly in movement modulation from the cortex to the nuclei and from these back to the motor cortex via thalamic nuclei. It has been shown that the caudate nucleus is heavily involved in learning and memory particularly in the treatment of feedback. Moreover, the limbic lobe contains the circunvolution of callous body, the subcallous circunvolution and the parahypocampal gyrus, being apparently the main responsable of emotional life, influencing the formation of memories, in association with the hyoptalamus, hypocampus, amígdala and other four brain areas. Overall the results obtained in the present study, lead us to postulate the existence of a correlation between high transcription levels of some DSCR genes in these structures and their potential role in the associated cognitive and emotional functions 28 ^,^ 29.

Previous reports showed that Calcineurin-nuclear factor of activated T cells (NFAT) signalling plays a critical role not only in the immune and nervous systems, but also in cardiovascular development and pathological endothelial cell activation during angiogenesis or inflammation 30 . The 1.5-fold increase in dosage of DSCR1 and DYRK1A cooperatively destabilizes a regulatory circuit, leading to reduced NFATc activity and many of the features of Down's síndrome. A mathematical modelling have predicted that autoregulation within of the pathway DSCR1, DYRK1A and NFATc accentuates the effects of trisomy, leading to failure to activate target genes under specific conditions 31 .

The brain homeostasis is measured by complex gene interactions that must be carried out between the great abundance and diversity of cells, which ultimately derives from the precise gene regulation in all structures. The results obtained in this study not only confirm it but extended the concept about designing a systemic model of global gene expression of nineteen genes located in the Down syndrome critical region in important structures of normal human brain that are controlling process of learning, movement and emotions. 
